# KSHV lytic proteins K-RTA and K8 bind to cellular and viral chromatin to modulate gene expression

**DOI:** 10.1371/journal.pone.0215394

**Published:** 2019-04-18

**Authors:** Rajeev Kaul, Pravinkumar Purushothaman, Timsy Uppal, Subhash C. Verma

**Affiliations:** 1 Department of Microbiology, University of Delhi South Campus, New Delhi, India; 2 Department of Microbiology and Immunology, University of Nevada, Reno School of Medicine, Reno, Nevada, United States of America; Wuhan University School of Life Science, CHINA

## Abstract

The oncogenic Kaposi’s sarcoma-associated herpesvirus (KSHV) has two distinct life cycles with lifelong latent/non-productive and a sporadic lytic-reactivating/productive phases in the infected immune compromised human hosts. The virus reactivates from latency in response to various chemical or environmental stimuli, which triggers the lytic cascade and leads to the expression of immediate early gene, i.e. Replication and Transcription Activator (K-RTA). K-RTA, the latent-to-lytic switch protein, activates the expression of early (E) and late (L) lytic genes by transactivating multiple viral promoters. Expression of K-RTA is shown to be sufficient and essential to switch the latent virus to enter into the lytic phase of infection. Similarly, the virus-encoded bZIP family of protein, K8 also plays an important role in viral lytic DNA replication. Although, both K-RTA and K8 are found to be the ori-Lyt binding proteins and are required for lytic DNA replication, the detailed DNA-binding profile of these proteins in the KSHV and host genomes remains uncharacterized. In this study, using chromatin immunoprecipitation combined with high-throughput sequencing (ChIP-seq) assay, we performed a comprehensive analysis of K-RTA and K8 binding sites in the KSHV and human genomes in order to identify specific DNA binding sequences/motifs. We identified two novel K-RTA binding motifs, (i.e. AGAGAGAGGA/motif RB and AGAAAAATTC/motif RV) and one K8 binding motif (i.e. AAAATGAAAA/motif KB), respectively. The binding of K-RTA/K8 proteins with these motifs and resulting transcriptional modulation of downstream genes was further confirmed by DNA electrophoretic gel mobility shift assay (EMSA), reporter promoter assay, Chromatin Immunoprecipitation (ChIP) assay and mRNA quantitation assay. Our data conclusively shows that K-RTA/K8 proteins specifically bind to these motifs on the host/viral genomes to modulate transcription of host/viral genes during KSHV lytic reactivation.

## Introduction

Infectious agents account for approximately 15% of all human cancers per year worldwide [1–4]. Kaposi’s sarcoma-associated herpesvirus or human herpesvirus-8 (KSHV/HHV-8), an oncogenic herpesvirus, is one of the seven DNA tumor viruses associated with several human malignancies. KSHV is the causative agent of Kaposi’s Sarcoma/KS, a common malignancy in HIV/AIDS patients, primary effusion lymphoma/PEL, and multicentric Castleman’s disease/MCD [5, 6]. KSHV’s life cycle involves prolonged, persistent latent phase and a short-lived lytic reactivation. During latency, the quiescent state of KSHV’s life cycle, only a limited set of viral genes/latent genes are expressed with no virion production. The virus can however reactivate from latency in response to different stimuli resulting in the expression of >80 genes culminating in the progeny infectious virions [7].

KSHV lytic DNA replication initiates at the lytic origin of replication (ori-Lyt) and requires both viral as well as cellular trans-acting elements. Two ori-Lyts (i.e. ori-Lyt-L/R) have been identified in the KSHV genome and KSHV viral K-RTA and K8 proteins have been demonstrated as ori-Lyt-binding proteins. K-RTA, also referred to as the latent-to-lytic master switch protein, is an immediate-early protein that activates the early/E and late/L lytic genes by transactivating multiple viral promoters. Multiple studies have shown that K-RTA is a direct transactivator of specific viral promoters even in the absence of other viral lytic genes [8]. Also, it is essential and sufficient to switch latent KSHV virus into the productive lytic phase to generate infectious progeny virions [9]. Another IE-gene that has been identified to be crucial for lytic reactivation is the K8/b-ZIP family protein, which is encoded by KSHV ORF8 [10]. KSHV K8 is the analogue of Epstein-Barr virus/EBV Zta protein. Though K8 had not been associated with transcriptional activation, it has been shown to interact with K-RTA and repress the K-RTA-mediated transcription of viral delayed-early genes [11–13]. K-bZIP is quintessential for KSHV lytic DNA replication during primary infection, but not during the lytic reactivation [13, 14]. Studies demonstrated that K-bZIP is a multifunctional protein that activated the transcription of specific KSHV and cellular promoters [15, 16]. Recently, K8 has been reported to coordinate with a noncoding T1.4 RNA to regulate KSHV lytic DNA replication during primary infection [17]. Studies have investigated the roles of these origin-binding proteins in KSHV infection and elucidated their effects on KSHV life cycle events including their recruitment and transactivation potential during viral reactivation. However the detailed DNA-binding profile of these proteins in the viral and host genomes remains elusive [15]. Guito and Lukac (2011) comprehensively discussed the studies describing the role of various stimuli and the regulatory mechanisms, which controls the functions of K-RTA [18].

In this study, we performed a chromatin immunoprecipitation assay coupled with high throughput sequencing (ChIP-seq) to comprehensively characterize the binding sites of these two DNA binding proteins, K-RTA and K8 of KSHV onto the viral and host genomes. These binding sites were further confirmed by ChIP-quantitative real-time PCR analyses. The identified host and viral binding sites were analysed to discover the motifs specific for K-RTA or K8 bindings. Furthermore, the ability of K8 and K-RTA to bind to these motif sequences and modulate transcription of downstream ORFs was confirmed by reporter promoter assay and DNA electrophoretic mobility shift assays. Finally, the effect of K-RTA and K8 on transcription of set of six representative host genes containing the discovered motifs in their promoter was analysed. Overall, our findings revealed novel viral and cellular DNAs associated with K-RTA and K8 proteins during lytic DNA replication that could be further exploited to understand the mechanisms of KSHV reactivation and associated tumorigenesis.

## Material and methods

### Constructs and cell lines

HEK293 cells were purchased from ATCC and maintained in Dulbecco’s modified Eagle medium (DMEM) supplemented with 10% fetal bovine serum (FBS, Atlanta Biologicals), 2 mM L-glutamine, 25 U/mL penicillin, and 25 μg/mL streptomycin. The KSHV-positive/BCBL-1 and KSHV-negative/BJAB cells were maintained in RPMI medium supplemented with 10% FBS, 2 mM L-glutamine, 25 U/mL penicillin, and 25 μg/mL streptomycin. TRExBCBL-1/RTA cells were provided by Dr. Jae Jung (University of Southern California) and were maintained in RPMI supplemented with 10% FBS, 2 mM L-glutamine, 25 U/mL penicillin, 25 μg/mL streptomycin, and 20 μg/mL hygromycin B. The iSLK RGB (containing KSHV BAC16) cells were provided by Dr. Jae Jung and were maintained in DMEM with 10% FBS, 2 mM L-glutamine, 25 U/mL penicillin, 25 μg/mL streptomycin, 600 μg/mL hygromycin B, 1 μg/mL puromycin and 250 μg/mL G418. The pLVX-GFP-RTA and pXi-K8-FLAG plasmids were described previously. The reporter vector, pGL4.23 luc2/minP for determining the activity of a minimal promoter was purchased from Promega (Promega Corporation). The sequences of K-RTA binding to the viral genome (RV) was cloned into pGL4.23 luc2/minP to obtain pGL4.23-RV. Similarly, K-RTA binding on cellular genome (RB) and K8 binding region (KB) was cloned into pGL4.23 luc2/minP to obtain pGL4.23-RB, and pGL4.23-KB. The integrity of the clones were confirmed by sequencing. K-RTA and K8 antibody were obtained from Dr. Izumiya (University of California, Davis) and Dr. Rossetto’s laboratory (University of Nevada, Reno), respectively. The mouse anti-control IgG antibody (ChIP grade # C15400001-15) was purchased from Diagenode Inc.

### Chromatin immunoprecipitation (ChIP) and sequencing (ChIP-seq)

Approximately, 20 million TRExBCBL-1/RTA induced with doxycycline for 24 hours (h) were harvested and fixed using 1% formaldehyde for 10 mins at room temperature, followed by addition of glycine at a final concentration of 125 mM for 5 mins to block the cross-linking. The cells were rinsed three times with ice-cold PBS and lysed in cell lysis buffer (5 mM PIPES, pH 8.0, 85 mM KCl, and 0.5 mM NP-40) supplemented with protease inhibitors for 10 mins on ice. The nuclei were enriched by low speed centrifugation and the obtained pellets were resuspended in nuclear lysis buffer (50 mM Tris-HCl, pH 8.1, 10 mM EDTA and 1% SDS, supplemented with protease inhibitors). Chromatin was sonicated to an average length of 500 to 800bp and centrifuged for 10 mins at 13,000 rpm to remove the cell debris. Samples were diluted 6-fold with ChIP dilution buffer (12.5 mM Tris, pH 8.0, 200 mM NaCl, and 1% Triton X-100) and pre-cleared with Protein A/G sepharose beads (GE Biotechnology) pre-treated with 1 mg/mL BSA and 1 mg/mL sheared salmon sperm DNA (GE Biotechnology) at 4°C for 30 mins. For each immunoprecipitation, the lysate was incubated with 10 μL of each antibody at 4°C overnight. No-antibody and antibody-isotype control immunoprecipitations were also performed. Protein A/G sepharose beads were blocked with 10 μg of sheared salmon sperm DNA and BSA at 4°C overnight and then washed with ChIP buffer. The blocked and washed protein A/G sepharose beads were incubated with the lysate at 4°C for 2 h. The beads were washed once with low-salt buffer (0.1% SDS, 0.1% Triton X-100, 2 mM EDTA, 20 mM Tris, pH 8, and 150 mM NaCl), once with high-salt buffer (0.1% SDS, 0.1% Triton X-100, 2 mM EDTA, 20 mM Tris, pH 8, and 500 mM NaCl), once with LiCl buffer (0.25 M LiCl, 1% NP-40, 1% deoxycholate, 1 mM EDTA, and 10 mM Tris pH 8), and twice with Tris-EDTA. Beads were then resuspended in TE, incubated with RNase A at 37°C for 30 mins, treated with proteinase K/10% SDS at 37°C for 4 h, followed by incubation at 65°C overnight. For input control samples, NaCl was added to the sonicated lysate to a final concentration of 0.3 M and incubated at 65°C overnight. The antibody, no-antibody, and isotype control immunoprecipitated samples were extracted with phenol-chloroform and precipitated using ethanol. The enriched DNA was quantified using a Qubit spectrophotometer and 10 ng of total DNA was used with the ChIP-seq DNA library preparation kit (Bioo Scientific), according to the manufacturer’s instructions. The ChIP-seq libraries were validated using a Bioanalyzer 2100 with a high-sensitivity DNA ChIP (Agilent) and quantified by qPCR (Kappa kit). ChIP-seq libraries were sequenced using a MiSeq (Illumina). The resulting sequencing data were analysed for determining the ChIP peaks using CLC Workbench (CLC Bio).

### Electrophoretic mobility shift assay (EMSA)

Nuclear extracts containing K-RTA and K8 from HEK293 cells were prepared by transiently transfecting K-RTA and K8 expression plasmids. Binding of these proteins to DNA were evaluated by EMSA using ^32^P-labeled probes containing specific K-RTA or K8 binding motifs sequences. The probes were synthesized as double-stranded DNA containing three copies of motif sequences in tandem (RV: 5’-AGAAAAATTCAGAAAAATTCAGAAAAATTC-3’, RB: 5’-AGAGACAGGAAGAGACAGGAAGAGACAGGA-3’, KB: 5’-AAAAATAAAAAAAAATAAAAAAAAATAAAA-3’). These probes were end-labeled with α-^32^P and purified on a GE Illustra ProbeQuant G-50 micro column (GE Healthcare, USA). A 50 μl DNA-protein binding assay mix containing 12 mM HEPES (pH 7.9), 12% glycerol, 60 mM KCl, 5 mM MgCl_2_, 0.12 mM EDTA, 0.3 mM DTT, 0.1 μg poly (dI/dC), and 0.05 μg of denatured salmon sperm DNA as carrier was used for EMSA. For detecting the super shift, K-RTA or K8 nuclear extracts were incubated with anti K-RTA or anti-K8 antibody along with the labeled probe. The reaction mix was incubated on ice for 30 mins and the bound complexes were resolved on 5% non-denaturing polyacrylamide gel in 0.5X TBE buffer (0.045 M Tris Borate, pH 8.2, and 1.0 mM EDTA). The gel was run at 4°C at 100V for 10–12 h and then exposed overnight on a phosphoimager screen. The signals were detected by autoradiography (GE Healthcare, Pittsburgh, PA).

### Dual luciferase reporter assay

HEK293 cells were transiently co-transfected using appropriate pGL4.23, internal control pRL-TK Renilla luciferase-expressing plasmid constructs (Promega), and expression vectors for eukaryotic expression of K-RTA or K8. The 4 μg of DNA was used per sample and all the transfections for reporter assays were done using metafectene (Biontex Laboratories GmbH), according to the manufacturer’s protocol. Following 48 h post-transfection, cells were harvested, lysed in cell lysis buffer (Promega) and 50 μl of the cell lysate was used for the reporter assay using dual luciferase reporter assay kit (Promega, USA). The relative luciferase activity was calculated by normalizing K-RTA and K8 luciferase activity to that of control Renilla luciferase activity to account for the transfection efficiencies. The results were plotted as a percentage of the activity to that of the empty pGL4.23 vector. A portion of the cell lysates was used for western blotting to detect K-RTA, K8 and GAPDH. All experiments were repeated multiple times, and the data shown are means of three independent experiments.

### Semi-quantitative real-time PCR

For quantitative Reverse-Transcriptase PCR assays, total mRNA from the cells was extracted using Illustra RNA spin Mini kit (GE Healthcare), according to the manufacturer’s instruction and cDNA was made using High-Capacity cDNA reverse-transcription kit (Applied Biosystems, USA). Each PCR reaction consisted of 10 μl of 2X SYBR GREEN Universal master mix (Bio-Rad Laboratories, USA), 5 μl of forward and reverse primers (0.5 μM), and 5 μl of the sterile water diluted cDNA. The qRT-PCR was performed to analyse the expression of RAB2A, NAP1L1, HDX, MBL2, SLITRK3, DSERG1, COL4A3BP, DMBT1, CDC7, MAGEC3, UBE3A and ROCK1P1 on an ABI Step One plus real-time PCR instrument (Applied Biosystems, USA) and relative gene copies or the transcripts were calculated by the ΔΔ*C*_*T*_ method as described earlier [19]. Table 1 lists the primers used for the amplification of target gene sequences. Each experiment included duplicate samples and the data shown represents the mean of three independent experiments.

**Table 1:** The primer sequences used in the ChIP-PCR analyses for determination of viral and host target genes downstream of K-RTA and K8.

Primer Name	Sequence
**K8 viral target genes**	
ORF6-F	AGCAGCGTGACACTACTA
ORF6-R	GTCTTGTATTGGTCGGTATCTT
K8-F	ATCTGGTTGATTGTGACTA
K8-R	TTACACGCCAGGTTATAG
K12-F	GCACGATAACCATACATG
K12-R	CGTCCAAATATGCCAAAT
**K-RTA viral target genes**	
K4.1-F	GACTGAAAAGGTCATAGAGAGGA
K4.1-R	TTCTCGGAAAGCCGGTGG
K12-F	GCACGATAACCATACATG
K12-R	CGTCCAAATATGCCAAAT
ORF59-F	TTAAATAGAACTTTTAGGGGAGGT
ORF59-R	GTGTGTGACTGACGATTTG
**K8 Host target genes**	
COL4A3BP-F	TTGTTACACTTGTTCCTAAG
COL4A3BP-R	CAGGAGAATTGCTTGAAC
DMBT1-F	CTTAATAATTACAAGTCACAAACA
DMBT1-R	CGATCCTATTCTCATCACAT
CDC7-F	CTGACACCTCCTGCTTAA
CDC7-R	GCTCGCTTGATCTTGATT
MAGEC3-F	TAGAATGAGACACCAACTG
MAGEC3-R	TAATGTGAGCAATCAATGTATT
UBE3A-F	TTACTCTTATTATCCTCCAA
UBE3A-R	CACATCAGGAAGCTATAC
ROCK1P1-F	GGACATTGTGACATATCT
ROCK1P1-R	CCTAGACAAGAGTTATATCA
**K-RTA Host Target genes**	
RAB2A-F	GATGAACTTGTGTTGGTAGAA
RAB2A-R	GGACATCTTCCAAGACTCT
NAP1L1-F	GCATACCTCCAGTCCTTAT
NAP1L1-R	ATGACAGACAAGCCAGTT
HDX-F	GCTCCTGAATCATCCATAA
HDX-R	CAATTATCAACTTAGAGACACAT
MBL2-F	TTTCTTTCGGTTTCAATAG
MBL2-R	CCTAATGTCATCAGTTCT
SLITRK3-F	GCTCTTCCACCAACATTA
SLITRK3-R	CAATAATCTACCATTCCAAGTT
DSERG1-F	ATGCTTTAAATCATGGGAAA
DSERG1-R	GGCTGTTTGAAATCAGTG

### ChIP-qPCR

Briefly, 20 million TRExBCBL-1 RTA and iSLK RGB cells were induced with doxycycline or doxycycline and sodium butyrate, respectively, for 24 h. The cells were subjected to cross-linking with 1% formaldehyde followed by re-suspending them in the chromatin shearing buffer after washing with PBS. Chromatin Immunoprecipitation was performed using the iDeal ChIP-qPCR kit for transcription factors (Diagenode Inc.). The ChIP enriched DNA was subjected to qRT-PCR, performed with 10 μl of 2X SYBR GREEN Universal master mix (Bio-Rad Laboratories, USA), 5 μl of forward and reverse primers (0.5 μM), and 5 μl of the sterile water diluted ChIP-enriched DNA. Specific primers corresponding to the region of RTA and K8 binding sites on viral and host genomes were used, which are listed in Table 2. The qRT-PCR was performed on an ABI Step One plus real-time PCR instrument (Applied Biosystems, USA) and enrichments of individual genomic regions were calculated as relative folds to that of anti-IgG, serving as a negative control as described earlier [19]. Each experiment included duplicate samples and the data shown represents the mean of three independent experiments.

**Table 2:** The primer sequences used in the RT-PCR analyses for determination of host target genes downstream of K-RTA and K8.

Primer Name	Sequence
**K8 target genes**	
COL4A3BP-F	GCCATTGAACAGCACAAGAC
COL4A3BP-R	GAGGTGGATGTTGCAGAGTAG
DMBT1-F	TGCTCAGCCACCCAAATAA
DMBT1-R	TGTCCCACTGGCATAGAATAAG
CDC7-F	TCAAACACCTCCAGGACAATAC
CDC7-R	GTACCTCATTCCAGCCTTCTAAA
MAGEC3-F	GCGAGCCCTTGTTCACTTAT
MAGEC3-R	TTCATCTGCATCTCTGCTCTTG
UBE3A-F	GGAAATCAGGAGAACCTCAGTC
UBE3A-R	CATTTCCACAGCCCTCAGTTA
ROCK1P1-F	TACAGGAGGTATTGTGAA
ROCK1P1-R	TAGGCAAGAGTTACATCA
**K-RTA Target genes**	
RAB2A-F	CAACACATGCAGGCAATCAG
RAB2A-R	GTTGGGCAGCTAGACAGTAAA
NAP1L1-F	GCTGTTCTCTATCAGCCTCTATTT
NAP1L1-R	CATCTTCTTCATCTGGTTTCCATTC
HDX-F	TGGATTGGGAATCGAAGAAGG
HDX-R	CCTGGTGTGAGTGCAGATAAA
MBL2-F	TCAGAATCTCATCAAGGA
MBL2-R	TTGTGTAGGTCAGTCTAT
SLITRK3-F	ATAATTCTTCTAAGCACAAT
SLITRK3-R	CTCGGTAATCTGACTAAT
DSERG1-F	ATAGAAGAATCACTAACTCA
DSERG1-R	GATACTAAGATGCTGTCA

### Accession numbers

The next generation sequence data of ChIP-seq data for RTA and K8 are available at the NCBI GenBank under the accession numbers GSE82283.

## Results

### Identification of KSHV K-RTA binding sites in the KSHV and human genomes

**Fig 1:**
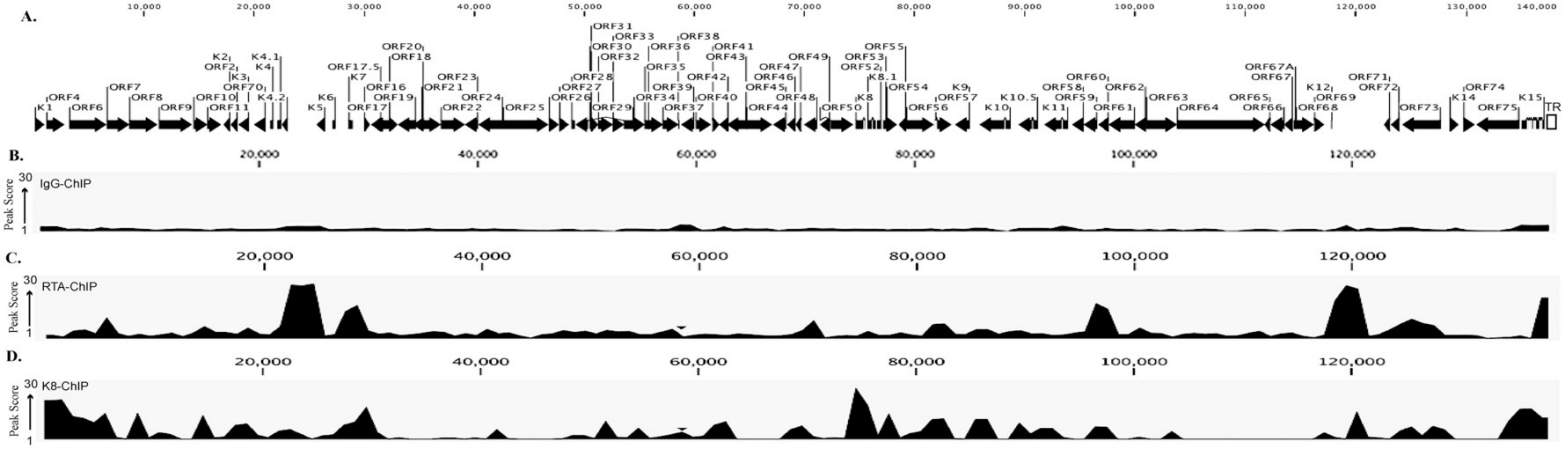
K-RTA and K8 binds to multiple regions of the KSHV genome identified through ChIP-seq. **(A)**. KSHV genome schematic with ORFs. **(B)**. ChIP-seq with control antibody, IgG was mapped to the KSHV genome. **(C)**. ChIP-seq with anti-K-RTA antibody mapped to the KSHV genome. **(D)**. ChIP-seq with anti-K8 antibody mapped to the KSHV genome. Relative binding of these proteins are represented by peak score and high peak score represents stronger binding.

**Fig 2:**
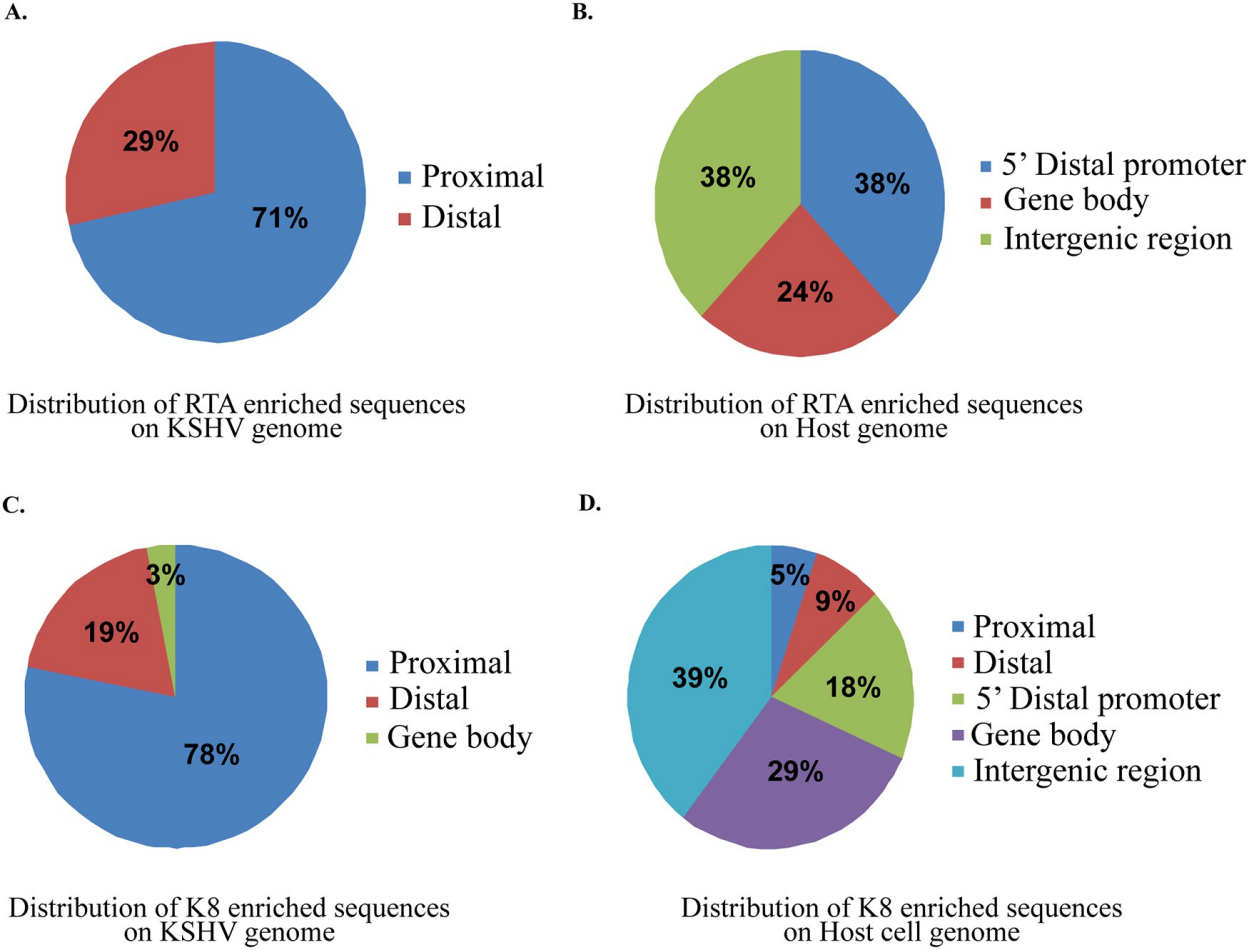
Relative distribution of K-RTA and K8 enriched sequences on KSHV as well as cellular genome identified through ChIP-seq. **(A)** Analysis of the K-RTA binding sites on the KSHV genome identified 71% of K-RTA binding sites in the proximal promoter region, whereas remaining 29% in the distal promoter region. **(B)** Mapping of the K-RTA binding site on the cellular genome identified majority of the enriched sequence in the intergene region (38%) or within the gene body (24%), while remaining 38% in the distal promoter region. **(C)** Relative distribution of K8 enriched sequence on the KSHV genome identified 78% of K8-enriched sequence in the proximal promoter region with 19% being in the distal promoter region and 3% in the gene body. **(D)** Analysis of the distribution of K8 enriched sequences on the host genome showed that most of the enriched sequences were in intergene region (39%) or within the gene body (29%), with the remaining 32% in the 5’ distal promoter region, comprising of both proximal (9%) and distal (5%) promoters.

In order to identify the genome-wide K-RTA binding sites on the KSHV and human genomes, chromatin immunoprecipitation (ChIP) with K-RTA and next generation sequencing (ChIP-seq) of the DNA was performed on KSHV-infected tetracycline-inducible TRExBCBL-1/RTA stable cells. The lytic cycle was triggered by treating the cells with doxycycline (1μg/ml) for 24 h and performing ChIP using anti-K-RTA antibody. The purified ChIP DNA was used for making ChIP-seq libraries using ChIP-seq kit (Bioo scientific). The libraries were sequenced using MiSeq (Illumina) and the sequences were mapped to the reference KSHV genome using ChIP-seq analysis tool of the CLC Genomic Workbench (CLC Bio). The resulting sequencing reads were mapped to the reference KSHV genome (NC_009333) and reference human genome (hg19) using CLC Genomics Workbench software. The ChIP-seq analysis was performed using reads from the input samples as reference in the analysis. Isogenic control antibody, IgG was used in the ChIP assay and the reads were also mapped to the viral genome. Importantly, K-RTA ChIP showed specific peaks on the viral genome as compared to the control antibody (IgG) with some previously known sites including the ori-Lyt regions (Fig 1). Mapping of the K-RTA ChIP reads to the host cellular genome showed specific binding and a total of 21 K-RTA binding sites were identified on the host genome. K-RTA showed strong binding at 7 specific regions on the viral genome. Table 3 lists these sites along with the closest transcriptional start sites (TSS). Out of the K-RTA binding sites on KSHV genome, 71% were in proximal promoter region (-1 to +1 Kb from TSS) whereas 29% were in distal promoter region (-1 to -10 Kb from TSS). Out of all the K-RTA binding sites on host cell genome, majority were found either in the intergenic region (38%) or within the gene body (24%), whereas remaining were mapped to distal promoter region (38%) (Fig 2A and 2B).

**Table 3:** K-RTA enriched regions on KSHV genome.

Region	Length	Reads	5’downstream gene	3’ downstream gene
+(22970..23051)	81	269	K4.1	T1.5
+(24032..24128)	96	288	K4.2	PAN RNA
+(28711..28779)	68	166	K6	
+(96940..97005)	65	184	ORF59	
+(119039..119123)	84	307	K12	
+(120358..120468)	110	360	K12	
+(137208..137289)	81	428	K15	

Our results indicated that K-RTA binds to KSHV genomic DNA upstream of K4.1, K4.2, T1.5, K6, PAN RNA, ORF59, K12, and K15 (Table 3). To verify our data, we compared the K-RTA binding sites of viral genome identified in our study with those determined previously. K-RTA has been previously reported to interact with promoter regions upstream of K6, PAN RNA, ORF59, K12, and K15 [15]. In this present study, we identified 2 novel K-RTA binding sites at 22,970–23,051 nucleotide position (upstream of K4.1 and T1.5 on either side) and at 24,032–24,128 nucleotide position (upstream of K4.2). However, the functional significance of these transcripts is yet to be investigated. These novel sites are in the long unique region/LUR of the KSHV genome, which encodes for approximately 90 ORFs with complex gene expression patterns [15]. Importantly, 21 K-RTA binding sites on host genome were found to be spread over 11 different chromosomes (Table 4). Most of these sites were in intergenic region or within the gene body. However, a number of sites discovered to be enriched for K-RTA were in distal promoter region, and may be significant for regulation of downstream target genes.

**Table 4:** K-RTA enriched regions on host genome.

Chromosome	Region	Length	Reads	5’downstream gene	3’ downstream gene
1	+(193831535..193831756)	221	10	RPL23AP22	EEF1A1P14
2	+(92319933..92320118)	185	18	LOC391405	
16	+(20727629^20727629)	0	26	ACSM1	ACSM3
23	+(83946286^83946286)	0	50	HDX	TEX16P
23	+(137633654^137633654)	0	22	KRT8P6	LOC100129662
5	+(10689..10782)	93	23		LOC100128803
5	+(11167..11233)	66	39		LOC100128803
8	+(61406060^61406060)	0	60	LOC100505532	RAB2A
9	+(3848999^3848999)	0	77	DSERG1	LOC100287493
9	+(120767700^120767700)	0	38	RPL35AP22	
10	+(1575292^1575292)	0	23	IDI1	PFKP
10	+(42389189..42389198)	9	32		LOC642424
10	+(55582193^55582193)	0	151	MBL2	MTRNR2L5
11	+(63490191^63490191)	0	18	ATL3	C11orf84
11	+(100512247^100512247)	0	21		LOC440063
12	+(33120431^33120431)	0	33	RPL35AP27	ASS1P14
12	+(76634161^76634161)	0	2935	NAP1L1	
3	+(53197256^53197256)	0	11	PRKCD, RFT1	
3	+(109411917^109411917)	0	46	DPA4	
3	+(110773681^110773681)	0	20	LOC151760	PVLR3
3	+(165106604^165106604)	0	195	SLITRK3	

### Mapping and characterization of KSHV K8 binding sites in the KSHV and human genomes

We also performed ChIP-seq analysis to discover genome-wide binding sites of K8 on lytically active KSHV genome as well as on the host genome. The virus reactivation was triggered by treating the cells with doxycycline (1μg/ml) and cells were harvested after 24 h induction for the analysis. K8-associated chromatin was isolated by ChIP using K8-specific antibody and subjected to Illumina sequencing. The resulting sequencing data was then aligned with the KSHV genome (GenBank accession no (NC_009333) and the human genome hg19 using CLC software. The aligned sequences were then analysed for K8 enriched region on the viral and cellular genomes using input as reference. The comparison of ChIP peaks, K8 specific vs IgG control ChIP data revealed that K8 associates with multiple regions on the KSHV and host genomes (Fig 1). Our analysis identified 32 K8-binding sites on the KSHV genome upstream of 43 ORFs (Table 5), and 38 K8-binding sites on the host genome, spread over 16 different chromosomes (Table 6). Out of the K8-binding sites on KSHV genome, 78% were in proximal promoter region (-1 to +1 Kb from TSS) whereas 19% were in distal promoter region (-1 to -10 Kb from TSS), and 3% were in the gene body. Of the K8 binding sites on host cell genome, majority were either in the intergenic region (39%) or within the gene body (29%), whereas remaining 32% were in proximal or distal promoter region and may have important role in regulation of downstream target genes (Fig 2C and 2D).

**Table 5:** K8 enriched regions on KSHV genome.

Region	Length	Reads	5’downstream gene	3’ downstream gene
+(1100..1151)	51	7039		ORF4
+(2857..2915)	58	1574		ORF6
+(5127..5193)	66	1208		ORF7
+(8449..8489)	40	1598		ORF8
+(14362..14405)	43	1262		ORF10
+(19441..19481)	40	249	ORF2	
+(21206..21243)	37	375	ORF70	
+(28019..28056)	37	350	K6	K7, PAN RNA
+(28529..28574)	45	307		K7, PAN RNA
+(29323..29425)	102	3709		ORF16
+(30356..30393)	37	483		
+(32626..32669)	43	170	ORF17	
+(41405..41444)	39	585	ORF23	ORF25
+(51730..51781)	51	873	ORF28	ORF33
+(54192..54235)	43	691	ORF28	ORF34
+(62042..62107)	65	1715	ORF39	ORF44
+(69554..69608)	54	592	ORF46	
+(74785..74873)	88	2693	ORF49	K8
+(75647..75705)	58	911	ORF49	K8.1
+(77585..77650)	65	1268	ORF52	ORF54
+(81994..82057)	63	1037	ORF55	ORF57
+(85984..86051)	67	985	vIRF-1	
+(89472..89517)	45	1193	vIRF-4	
+(91954..91998)	44	426	vIRF-3	
+(96966..97003)	37	705	ORF59	
+(103616..103642)	26	236	ORF62	ORF64
+(119212..119250)	38	75	K12	
+(120327..120401)	74	1586	K12	
+(127817..127840)	23	615	ORF73	K14
+(134856..134914)	58	1727	ORF75	
+(136344..136477)	133	7040	ORF75	
+(137663..137680)	17	150	K15	

**Table 6:** K8 enriched regions on host genome.

Chromosome	Region	Length	Reads	5’downstream gene	3’ downstream gene
1	+(42686696^42686696)	0	101	GUCA2A	
1	+(91852914..91852946)	32	357		CDC7
1	+(121484115..121484124)	9	1631		
1	+(174811173^174811173)	0	90		
1	+(187481037^187481037)	0	50		FDPSP1
1	+(212760508^212760508)	0	80		FAM71A
2	+(47074577^47074577)	0	88		
2	+(89872109..89872110)	1	924	IGKV2-40	IGKV2D-40
2	+(92324430..92324492)	62	280	LOC391405	
2	+(133012659..133012728)	69	163		CDC27P1
15	+(25723069^25723069)	0	73	UBE3A	
15	+(57178141..57178291)	150	97		TCF12
16	+(25840633^25840633)	0	112	LOC100420641	MIR548W
16	+(33957201..33957226)	25	495		
16	+(33963336..33963357)	21	201	NCRNA00273	
16	+(46424214..46424240)	26	132		
18	+(108286..108348)	62	1097		ROCK1P1
18	+(46448369..46448439)	70	41		
19	+(27890187..27890188)	1	60	LOC100101266	
21	+(9827429..9827458)	29	208		SNX18P11
23	+(46598903^46598903)	0	66	LOC100420317	RP2
23	+(140829049^140829049)	0	81	SPANXD	MAGEC3
5	+(32514278^32514278)	0	60	MIR579, ZFR	LOC646616
5	+(74853605^74853605)	0	98	COL4A3BP	ANKDD1B
7	+(33282494^33282494)	0	91	RP9	LOC100421336
7	+(61085291..61085292)	1	124	LOC100420541	LOC100419991
7	+(61969238..61969244)	6	2547	LOC100420541	LOC100419991
8	+(70602415..70602464)	49	167		LOC100101127
9	+(3848999^3848999)	0	231	DSERG1,RFX3	SLC1A1
10	+(42396151..42396154)	3	1289		
10	+(55582193^55582193)	0	505	MBL2	LOC100420737
10	+(124312796^124312796)	0	61		DMBT1
11	+(39261585^39261585)	0	86		LOC100421559
11	+(55900116^55900116)	0	67	OR5BN2P	
12	+(76199928^76199928)	0	68	RPL10P13	
4	+(35094470^35094470)	0	57		SEC63P2
4	+(147963268^147963268)	0	82	TTC29	
4	+(182005058^182005058)	0	40	LOC100288337	LOC132386

K8 enriched regions on the KSHV genome were upstream to ORF2, ORF4, ORF6, ORF7, ORF8, ORF10, ORF70, K6, K7, K7, ORF16, ORF17, ORF23, ORF25, ORF28, ORF33, ORF34, ORF39, ORF44, ORF46, ORF49, K8, K8.1, ORF52, ORF54, ORF55, ORF57, vIRF1, vIRF4, vIRF3, ORF59, ORF62, ORF64, K12, ORF73, ORF75, and K15 (Table 5).

### K-RTA and K8 enriched on specific motif sequences on KSHV and host genome

**Fig 3:**
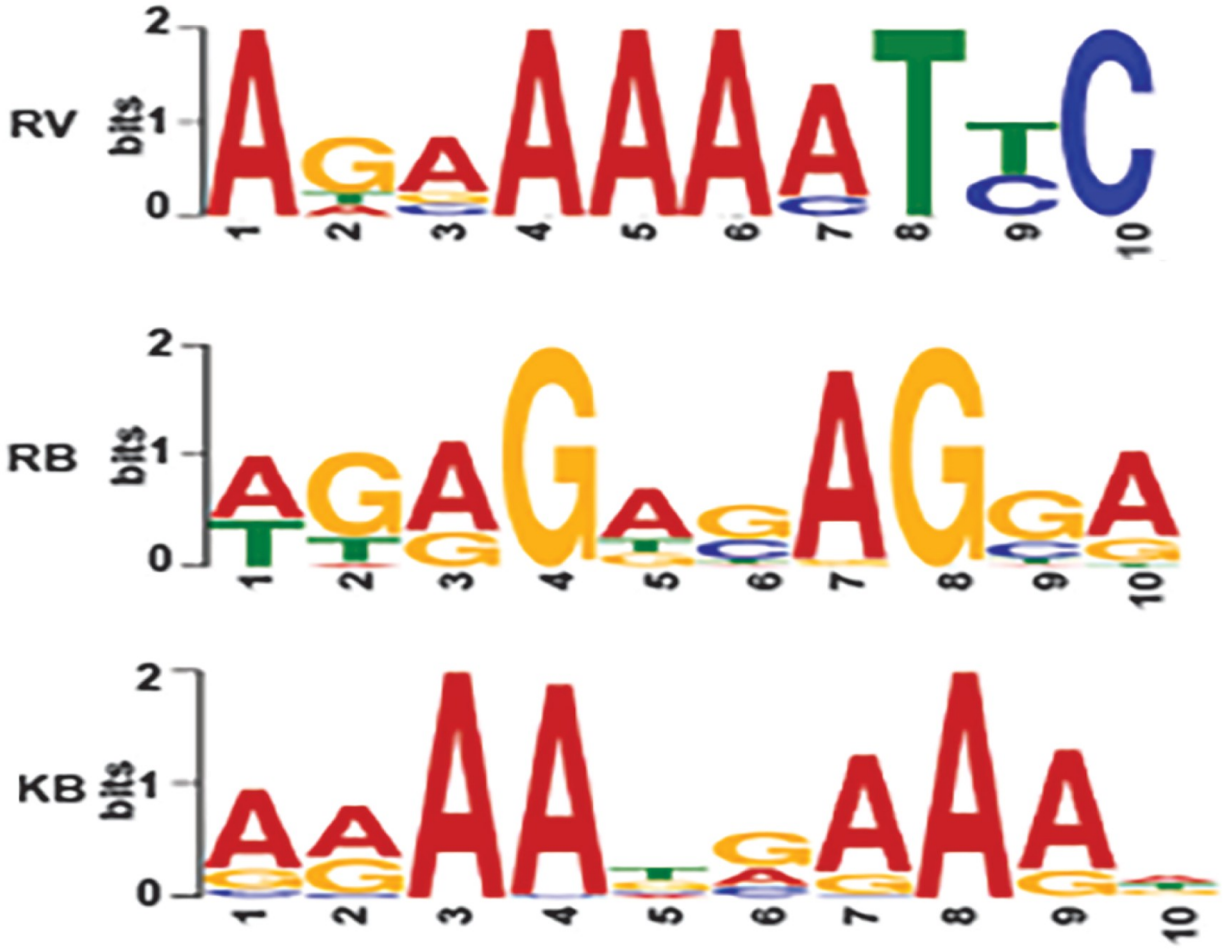
ChIP-seq identified novel K-RTA and K8 binding sequences on KSHV as well as on the cellular genome. Analysis of the K-RTA binding sites identified K-RTA specific motif AGAGAGAGGA (motif RB) with strong enrichment among the DNA sequences on both host and viral genome and K8-specific motif AGAAAAATTC (motif RV) with a strong central enrichment among the DNA sequences on viral genome bound by K-RTA. Additionally analysis of the K8 enriched sites showed a motif AAAATGAAAA (motif KB) which also has a strong enrichment among the DNA sequences on both host and viral genome.

To identify the DNA motifs driving the association of K-RTA to both the host and viral genomes, we analysed the viral and host genomic DNA sequences 250bp upstream and downstream from the centre of K-RTA binding peaks detected by ChIP-seq assay. The sequences were then submitted to MEME-ChIP analysis [20]. The viral sequences were analysed separately as well as in combination with host sequences and vice-versa. The analysis determined that one particular motif AGAGAGAGGA/motif RB had a strong central enrichment among the DNA sequences on both host and viral genomes bound by K-RTA. The analysis of motif RB using TOMTOM motif comparison tool revealed that it shows similarity with the Ets1 motif that has winged helix-turn-helix DNA binding domain [21]. Many different proteins with diverse biological functions contain a winged helix DNA-binding domain, including transcriptional repressors such as biotin repressor, LexA repressor and the arginine repressor [22]. Our results strongly suggested that K-RTA binds to the Ets1-like motif on viral DNA in KSHV-infected cells. Further analysis of ChIP-seq data using MEME ChIP analysis showed the presence of another motif, AGAAAAATTC/motif RV with a strong central enrichment sequence among the K-RTA bound sequences identified on the viral genome. The analysis of motif RB using TOMTOM motif comparison tool revealed it to be a novel motif without a significant similarity with any previously reported motif in JASPAR CORE database (Fig 3).

The analysis of 500bp sequences flanking the K8 enriched sites on viral and host genomes using MEME-ChIP revealed a motif AAAATGAAAA/motif KB containing strong central enrichment sequences among the DNA sequences on both host and viral genomes bound by K8. The analysis of motif KB using TOMTOM motif comparison tool revealed it’s similarity with PRDM1 motif, which belongs to the class of zinc coordinating domain motifs from beta-beta-alpha zinc finger family motifs (Fig 3).

### K-RTA and K8 associated with their binding motif sequences *in vitro*

**Fig 4:**
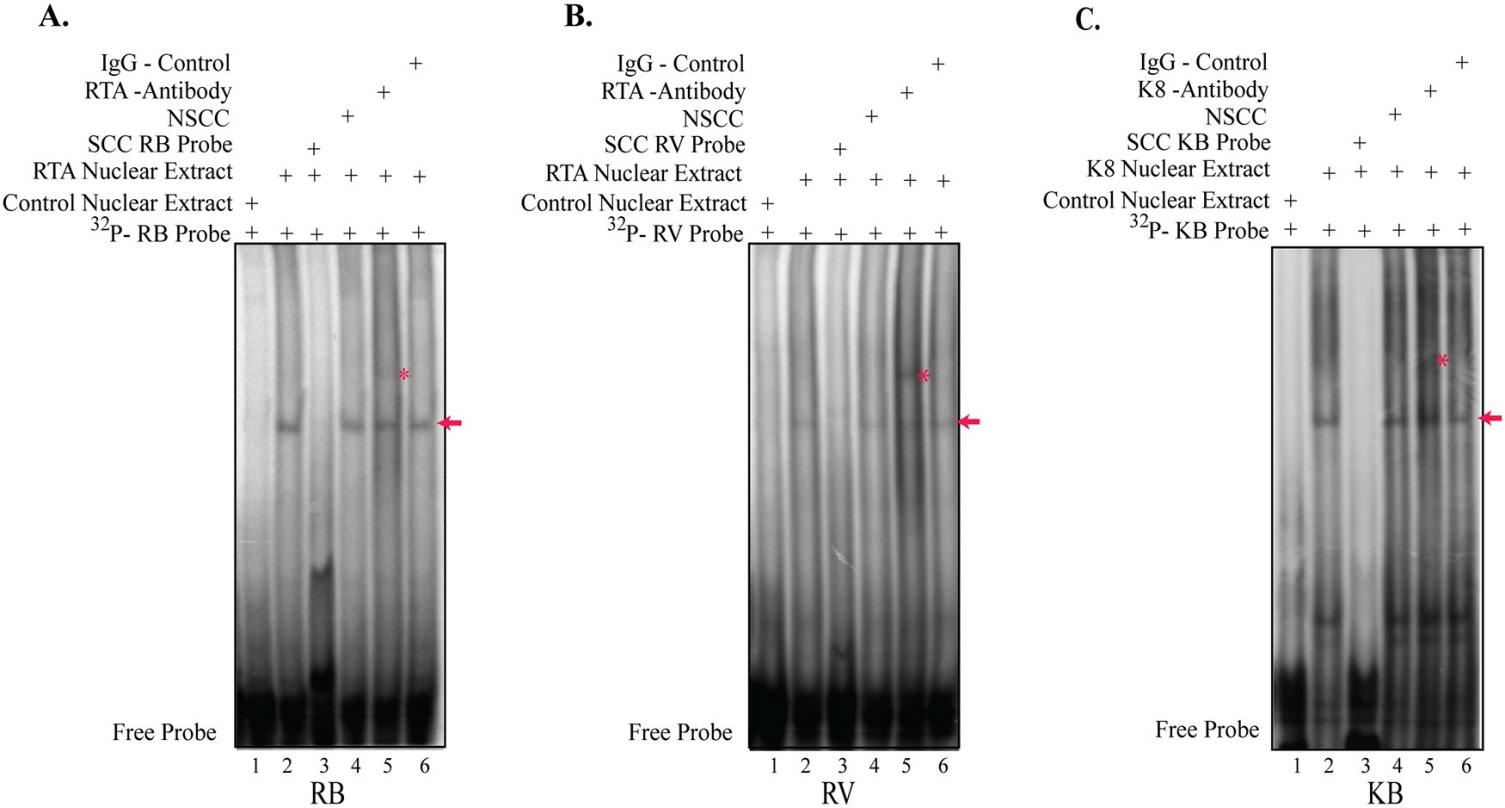
Electrophoretic mobility shift assay (EMSA) showing K-RTA and K8 binding to the novel sequence motifs. **(A)** EMSA showing K-RTA binding to the RB motif. EMSA was performed using ^32^P-labeled double-stranded K-RTA binding RB sequence probe with control nuclear extract (lane 1) or K-RTA nuclear extract showing binding to RB probe (lane 2), 200x specific cold competitor RB probe (SCC, lane 3); RB probe with 200x non-specific cold competitor GFP probe (NSCC, lane 4), K-RTA binding to RB probe along with specific antibody against K-RTA showing a super-shift (lane 5), and RB probe with control antibody which does not affect the K-RTA binding to RB probe (lane 6). **(B)** EMSA showing K-RTA binding to the RV motif. EMSA was performed using ^32^P-labeled double-stranded K-RTA binding RV sequence probe with control nuclear extract (lane 1) or K-RTA nuclear extract showing binding to RV probe (lane 2), 200x specific cold competitor RV probe (SCC, lane 3), RB probe with 200x non-specific cold competitor GFP probe (NSCC, lane 4), K-RTA binding to RV probe along with specific antibody against K-RTA showing a super-shift (lane 5), and K-RTA binding to RV probe along with control antibody which does not affect the K-RTA binding to RV probe (lane 6). **(C)** EMSA showing K8 binding to the KB motif. EMSA was performed using ^32^P-labeled double-stranded K8 binding KB sequence probe with control nuclear extract (lane 1), or K8 nuclear extract showing binding to KB probe (lane 2), 200x specific cold competitor KB probe (SCC, lane 3), KB probe with 200x non-specific cold competitor GFP probe (NSCC, lane 4), K8 binding to KB probe along with specific antibody against K8 showing a super-shift (lane 5), and K8 binding to KB probe along with control antibody which does not affect the K8 binding to KB probe (lane 6).

The preferential enrichment of K-RTA and K8 at novel motif sequences containing three copies of motifs RB (AGAGAGAGGA) or RV (AGAAAAATTC) or KB (AAAATGAAAA) in tandem repeats were further established by an electrophoretic mobility shift assay (EMSA). For EMSA, radiolabeled double-stranded RB, RV and KB motifs oligos were used as probe. Protein from nuclear extracts from HEK293 cells transiently transfected with the corresponding transient expression plasmids were evaluated by EMSA for their DNA binding ability, with HEK293 cells transfected with empty vector serving as a control. The radiolabeled RB, RV and KB probe bound to K-RTA and K8 proteins, respectively, and showed a specific shift probe mobility (Fig 4A–4C, lanes 2, shown by an arrow). However, the bindings of K-RTA and K8 to radiolabeled RB, RV and KB probe were significantly reduced by an addition of specific cold competitor, SCC (Fig 4A–4C, lanes 3), but not with a non-specific cold probe (non-specific cold competitor, NSCC) (Fig 4A–4C, lanes 4). Additionally, incubating the complex with specific antibody against K-RTA and K8 (Fig 4A–4C, lanes 5), but not with the control antibody, further retarded the mobility of the probe complex (Fig 4A–4C, lane 6), thus confirming specific association of K-RTA and K8 binding to the RB, RV and KB motifs. Taken together, our EMSA results clearly confirmed the ability of RB, RV and KB motifs to associate with KSHV K-RTA and K8 lytic proteins.

### K-RTA and K8 modulated transcription of gene downstream to their binding motifs

**Fig 5:**
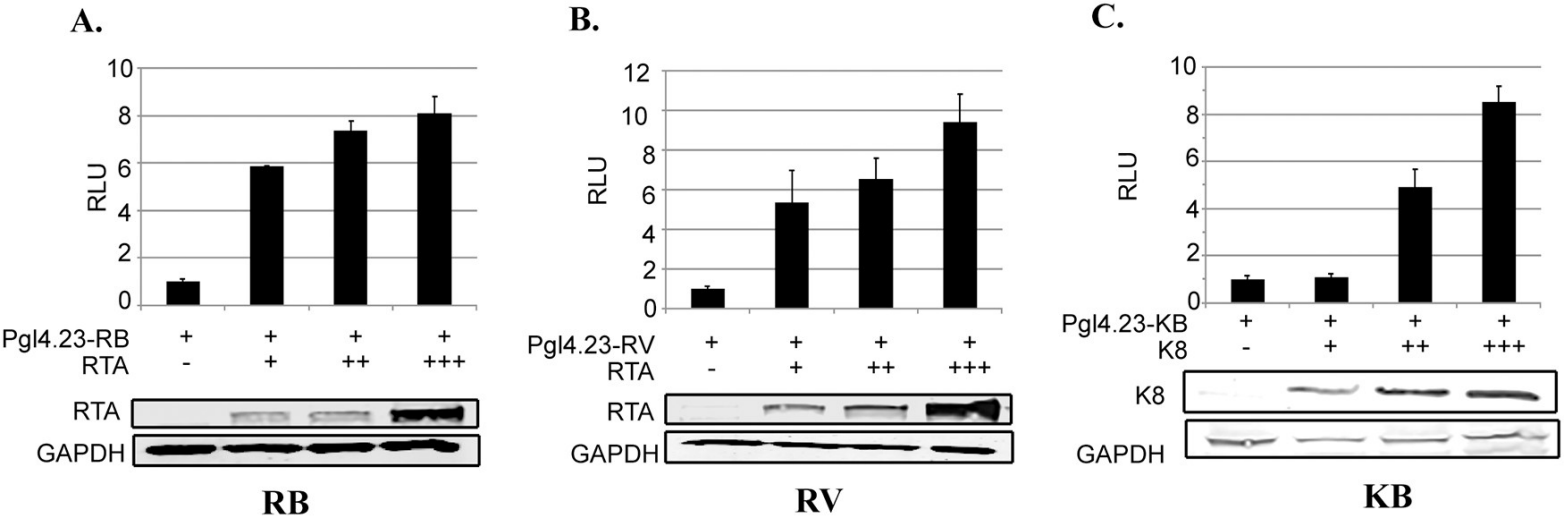
Luciferase reporter assay using minimal promoter sequence of K-RTA and K8 binding sequence. Relative fold increase of: **(A)** pGL4.23-RB, and **(B)** pGL4.23-RV luciferase activity in the presence of increasing amounts of K-RTA. Three copies of K-RTA binding motif RB or RV sequence was placed upstream of a minimal TATA promoter (minP) in a luciferase vector and transiently expressed in HEK293 cells along with the K-RTA protein. Relative luciferase units were calculated relative to the basal promoter activity. The lower panels are western blots showing relative expression of K-RTA and GAPDH from equal amounts of cell lysates from each experimental set. Relative fold increase of: **(C)** pGL4.23-KB luciferase activity in presence of increasing concentration of K8. Three copies of K8 binding motif KB sequence was placed upstream of a minimal TATA promoter (minP) in a luciferase vector pGL4.23-KB and transiently expressed in HEK293 cells along with K8. Relative luciferase units were calculated relative to the basal promoter activity. The lower panels are western blots showing relative expression of K8 and GAPDH from equal amounts of cell lysates from each experimental set.

To further investigate whether the enrichment of K-RTA or K8 at their binding motifs can modulate the transcription of downstream target genes, we cloned three copies of the binding motifs sequence in tandem upstream of a minimal TATA promoter (minP) in a luciferase vector encoding a synthetic firefly luciferase gene luc2 (pGL4.23, Promega, Madison, WI). This vector pGL4.23 carries minimal promoter (minP) with a TATA-box promoter element immediately upstream of the luc2 gene and immediately downstream of the multiple cloning sites. These are referred to as pGL4.23-RB, pGL4.23-RV, and pGL4.23-KB. HEK293 cells were co-transfected with DRA-Luc reporter plasmid, together with increasing concentrations of either K-RTA or K8 expression vectors. Dual luciferase assay was performed according to the manufacturer’s instructions (Promega, Inc). Our data showed that increasing amount of both K-RTA and K8 proteins resulted in an upregulation (up to 10 fold) of downstream reporter gene expression. The expression of corresponding amount of K-RTA and K8 in equal amount of lysates was also confirmed by western blot for K-RTA, K8 along with GAPDH as a loading control (Fig 5). These results clearly pointed out for a specific association of both these KSHV proteins, K-RTA and K8 to these motifs. This association in turn led to a transcriptional regulation of viral as well as host genes with specific sequence motifs in their promoter region by K-RTA (Fig 5A and 5B) as well as by K8 (Fig 5C).

### K-RTA and K8 modulated the transcription of viral and host genes downstream of these specific motifs

**Fig 6:**
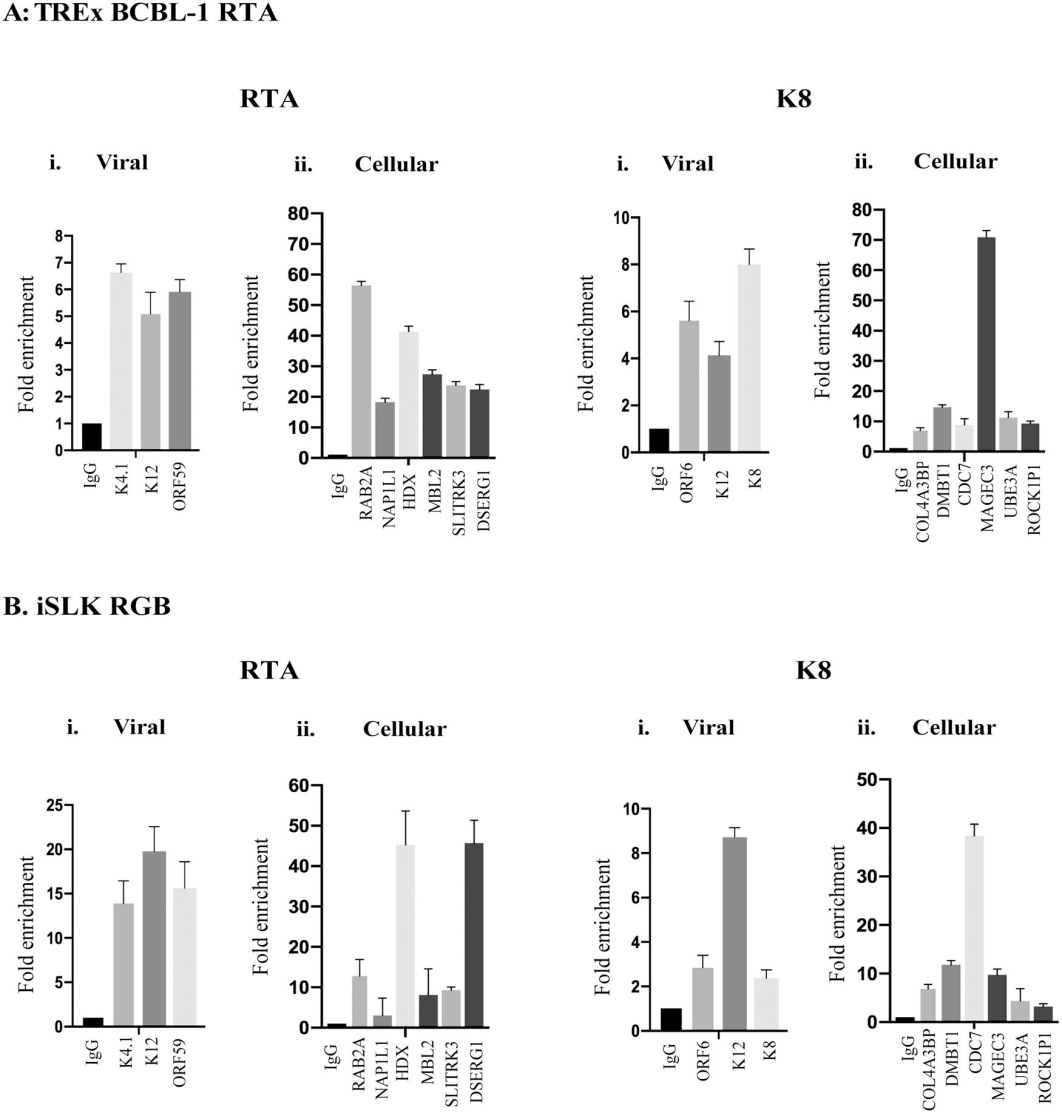
ChIP and quantitative PCR analyses of K-RTA and K8 binding sites identified using ChIP-Seq. The fold enrichment of K-RTA and K8 enriched viral and cellular genes were calculated relative to that of anti-IgG, serving as a negative control using: **(A)** TRExBCBL-1 RTA, and **(B)** iSLK RGB cells. Quantitative real time-PCR was performed to analyze the levels of K-RTA enriched viral (K4.1, K12, ORF59), cellular (RAB2A, NAP1L1, HDX, MBL2, SLITRK3, DSERG1), and K8 enriched viral (ORF6, ORF12, K8) as well as cellular (COL4A3BP, DMBT1, CDC7, MAGEC3, UBE3A, ROCK1P1) genes. The error bars represent standard deviations from the mean of at least three experimental replicates.

To further confirm the binding sites of K-RTA and K8, identified using ChIP-Seq, and to investigate their functional significance in the regulation of viral and host gene expression, we performed ChIP-qPCR analyses on TRExBCBL-1 and iSLK RGB cells. We selected 6 host genes each with K-RTA binding motifs (RAB2A, NAP1L1, HDX, MBL2, SLITRK3, DSERG1) and K8 binding motifs (COL4A3BP, DMBT1, CDC7, MAGEC3, UBE3A, ROCK1P1) identified from our ChIP-seq assay. We also chose three viral genes each with K-RTA binding motifs (K4.1, K12, ORF59) and K8 binding motifs (ORF6, K12, K8) in their promoter regions (Table 1). Relative binding of K-RTA to the viral genome in both the cell lines, determined by fold enrichment, showed K-RTA’s association with viral chromatin at previously known (K12 and ORF59) as well as at the newly identified sites from our ChIP assay (K4.1) (Fig 6A and 6B, RTA i-Viral). K-RTA binding on the cellular chromatin was also confirmed by the detection of K-RTA’s association on the chromatins of RAB2A, NAP1L1, HDX, MBL2, SLITRK3, DSERG1 promoter region in both the cell lines (Fig 6A and 6B, RTA ii-Cellular). Similarly, K8 showed binding to the viral as well as cellular chromatins of cellular targets identified from our ChIP-seq assay (Fig 6A and 6B, K8, i-viral and ii-Cellular).

**Fig 7:**
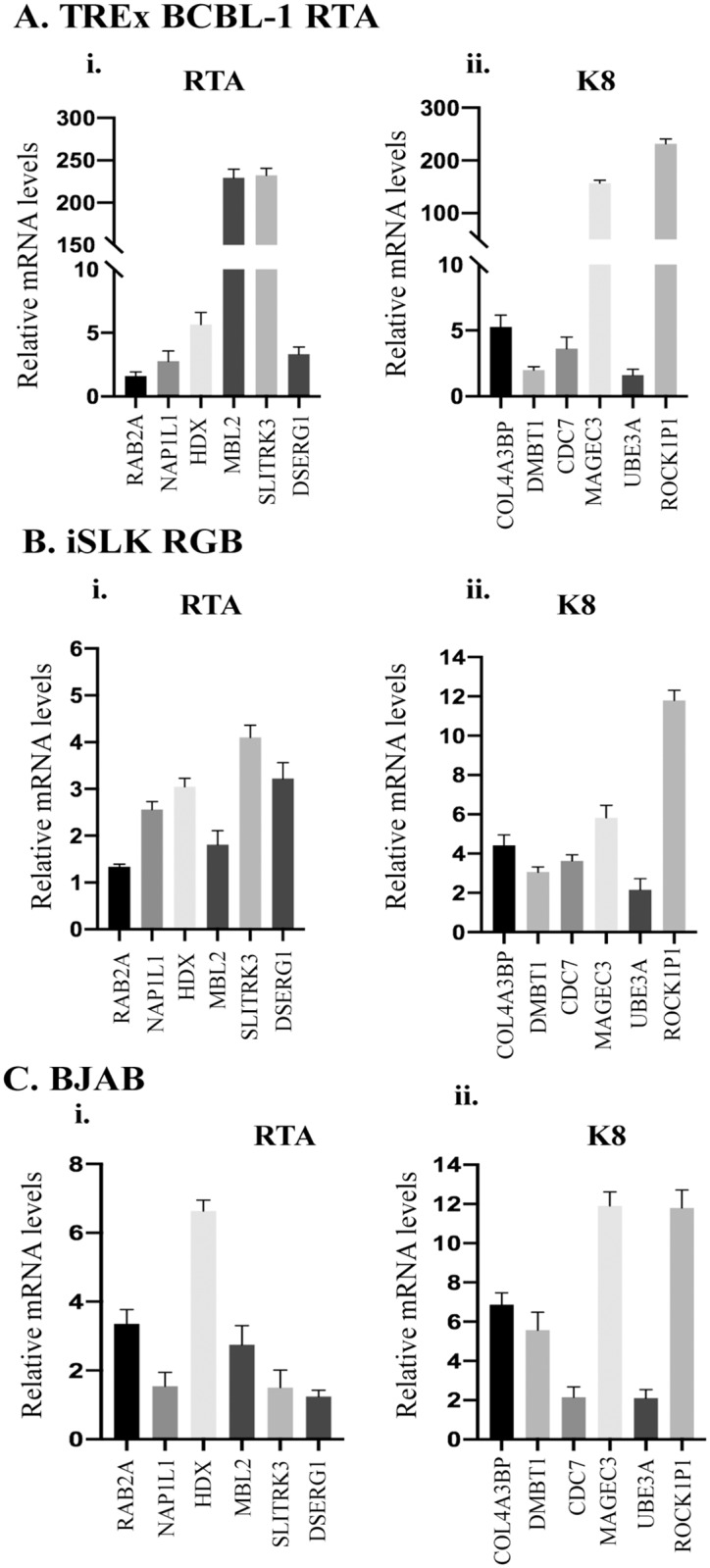
Relative mRNA levels of various K-RTA and K8 enriched host genes. The relative mRNA levels of K-RTA and K8 enriched cellular genes were analyzed using KSHV-positive: **(A)** TRExBCBL-1 RTA, and **(B)** iSLK RGB cells and **(C)** KSHV-negative BJAB cells stably expressing either K-RTA or K8. Quantitative real time-PCR was performed to analyze the expression levels of K-RTA enriched RAB2A, NAP1L1, HDX, MBL2, SLITRK3, DSERG1, and K8 enriched COL4A3BP, DMBT1, CDC7, MAGEC3, UBE3A, ROCK1P1 cellular genes, normalized with respect to ß-actin. The error bars represent standard deviations from the mean of at least three experimental replicates.

To confirm the role of K-RTA and K8 in regulation of host gene expression *via* their binding to their specific binding sites, we performed qPCR assays to detect the mRNA levels of genes, which were in proximity to the binding sites identified in our ChIP-seq assay. We selected those 6 representative cellular genes, used in ChIP-qPCR assay (Fig 6) to detect their mRNA levels using the primers listed in Table 2. As expected, the expression of K-RTA (Fig 7A and 7Bi) and K8 (Fig 7A and 7Bii) following reactivation of TRExBCBL-1 RTA and iSLK RGB cells led to an upregulation, although varying degrees, of these genes in both the cell lines tested. We further tested whether the expression of these cellular genes were independent of other viral factors during reactivation, we overexpressed K-RTA or K8 in a KSHV negative cell line, BJAB and compared their mRNA with vector transfected cells. Interestingly, in agreement with data from KSHV infected cells, the expression of K-RTA (Fig 7Ci) and K8 (Fig 7Cii) were able to upregulate those cellular genes confirming their role in controlling gene transcription.

## Discussion

KSHV reactivation is an extremely complex process that involves a combination of both viral and cellular factors. KSHV-encoded K-RTA is a master regulator for the lytic reactivation from viral latency. Expression of K-RTA and K-bZIP/K8 is essential for KSHV reactivation. Additionally, K8 directly binds to K-RTA through K-bZIP’s basic domain and a specific K-RTA region to modulate gene expression. To explore the genome-wide binding sites of K-RTA and K8 on KSHV and host cell genomes during KSHV reactivation, chromatin immunoprecipitation of K-RTA and K8 were carried out, followed by parallel sequencing (ChIP-seq) in doxycycline-induced KSHV-positive TRExBCBL-1/RTA stable cells. We identified 21 K-RTA binding sites and 38 K8 binding sites on host genome and 7 K-RTA binding sites and 32 K8 binding sites on the KSHV genome, respectively (Tables 3–6). Our data demonstrated that out of all the K-RTA binding sites identified on host cell genome, majority were either in intergenic region (38%) or within the gene body (24%), whereas remaining were in distal promoter region (38%). Similarly, for all the K-RTA binding sites on KSHV genome, 71% were found in proximal promoter region (-1 to +1 Kb from TSS) whereas 29% were mapped in distal promoter region (-1 to -10 Kb from TSS) (Fig 2A and 2B).

Additionally, K-RTA also binds to KSHV genomic DNA promoter regions upstream to K4.1, K4.2, T1.5, K6, PAN RNA, ORF59, K12, and K15 as well of K6, PAN RNA, ORF 59, K12, and K15 [15]. To further evaluate our data, we compared K-RTA binding sites on KSHV genome, identified in our study with a few previously identified sites. Interestingly, we found 2 novel K-RTA binding sites, one at 22,970–23,051 base position upstream to K4.1 and T1.5 on either side and one at 24,032–24,128 base position upstream to K4.2 (Table 3). However, the functional significance of these novel sequences in the modulation of KSHV transcripts is yet to be explored. Surprisingly, both these novel sites are in LUR of KSHV genome, which encodes for approximately 90 ORFs with complex gene expression patterns [15]. Furthermore, these 21 K-RTA binding sites on the host genome were spread over 11 chromosomes (Table 4). Most of these sites were in intergenic region or within the gene body (Fig 2B). However a good number of sites discovered to be enriched for K-RTA were in distal promoter region, and may be crucial for the regulation of downstream target genes.

Correspondingly, our K8 ChIP-seq analysis also showed 32 K8 binding sites upstream of 43 ORFs of KSHV (Table 5) as well as 38 K8 binding sites on host genome distributed over 16 chromosomes (Table 6). Out of the known K8 binding sites on KSHV genome, 78% were detected in the proximal promoter region (-1 to +1 Kb from TSS) whereas 19% were in distal promoter region (-1 to -10 Kb from TSS), and 3% were in gene body (Fig 2C). Of the K8 binding sites on the host cell genome, majority were either in intergenic region (39%) or within the gene body (29%), whereas remaining 32% were in proximal or distal promoter region (Fig 2D). Further our results showed that K8 is enriched on KSHV genomic DNA upstream to ORF2, ORF4, ORF6, ORF7, ORF8, ORF10, ORF70, K6, K7, K7, ORF16, ORF17, ORF23, ORF25, ORF28, ORF33, ORF34, ORF39, ORF44, ORF46, ORF49, K8, K8.1, ORF52, ORF54, ORF55, ORF57, vIRF1, vIRF4, vIRF3, ORF59, ORF62, ORF64, K12, ORF73, ORF75, and K15 (Table 5). However a good percentage of sites discovered to be enriched for K8 were in proximal and distal promoter regions, and probably play an important role in the regulation of downstream target genes.

Furthermore, our MEME-ChIP analysis [20] to evaluate the DNA motifs driving the association of K-RTA to the host and viral genome flanking sequences, 250bp upstream and downstream from the centre of K-RTA binding peaks, consistently identified AGAGAGAGGA/motif RB that had a strong central enrichment among the DNA sequences on both host and viral genomes bound by K-RTA as well as AGAAAAATTC/motif RV that had a strong central enrichment among the DNA sequences on viral genome bound by K-RTA (Fig 3). Interestingly, K-RTA binding motif identified through our ChIP-seq and MEME analysis is not identical to the previously identified motifs but have strong similarities in terms of A/T sequence in the core sequence [23–26]. The analysis of motif RB using TOMTOM motif comparison tool further revealed that it was a novel motif and did not show any similarity with any motif in JASPAR CORE database. Additionally our TOMTOM motif analysis also showed that motif RB has high similarity with Ets1 motif that has winged helix-turn-helix DNA binding domain [21]. These results strongly suggest that K-RTA binds to Ets1 like motif on viral DNA in KSHV infected cells. [22]. Similarly, our MEME-ChIP analysis of 500bp flanking sequences of the K8 enriched sites on viral and host genomes revealed motif AAAATGAAAA/motif KB had a strong central enrichment among the DNA sequences on both host and viral genomes bound by K8 (Fig 3). Subsequently, TOMTOM motif comparison analysis revealed that this motif shows similarity with PRDM1 motif, which belongs to the class of zinc coordinating domain motifs from beta-beta-alpha zinc finger family motifs.

To further study the interaction of K-RTA and K8 to these novel sequence motifs, we performed an electrophoretic mobility shift assay (EMSA). EMSA results consequently confirmed the preferential enrichment of K-RTA and K8 at novel motif sequences identified in our ChIP-seq analysis. Furthermore, our EMSA results consistently showed that K-RTA can directly bind to oligos containing RB or RV motif sequences, whereas K8 can directly bind to KB motif containing oligos. This was also confirmed by the super-shift observed using specific anti K-RTA and K8 antibodies. These data clearly substantiates the ability of RB, RV and KB motifs to bind to KSHV viral proteins K-RTA and K8, respectively (Fig 4).

In order to investigate whether enrichment of K-RTA or K8 at their binding motifs can modulate the transcription of downstream target genes, we performed a dual luciferase assay. Our data consistently showed that increasing amount of both K-RTA and K8 proteins led to an upregulation of luciferase reporter gene expression (Fig 5). The expression of corresponding amount of K-RTA and K8 were also further confirmed by western blot analysis (Fig 5).

To confirm the bindings of these two proteins on viral and cellular genomes, we selected gene promoters with K-RTA and K8 binding sites, identified in our ChIP assay and tested their association with these proteins. Our data clearly showed that both, K-RTA and K8 can efficiently bind to the chromatin of those identified regions confirming the validity of our ChIP-seq. In order to understand the functional significance of these interactions onto the viral and cellular genome, in the regulation of genes expression, we performed a qPCR assay on KSHV-positive TRExBCBL-1 RTA and iSLK RGB cells and on KSHV-negative BJAB cells stably expressing K-RTA or K8 proteins. Interestingly, all the selected host genes with K-RTA binding motifs (RAB2A, NAP1L1, HDX, MBL2, SLITRK3, DSERG1) and K8 binding motifs (COL4A3BP, DMBT1, CDC7, MAGEC3, UBE3A, ROCK1P1) in their promoter regions showed upregulation following expression of the indicated proteins (Fig 7). Taken together, our data showed that K-RTA and K8 specifically bind to specific sequence motif on host and viral genomes and modulates transcriptional regulation of viral as well as host genes during KSHV lytic reactivation.

## References

[1] Gencer S, Salepci T, Ozer S. Evaluation of infectious etiology and prognostic risk factors of febrile episodes in neutropenic cancer patients. J Infect. 2003;47(1):65–72. Epub 2003/07/10. . 12850165 10.1016/s0163-4453(03)00044-6

[2] O’Brien K, Cokkinides V, Jemal A, Cardinez CJ, Murray T, Samuels A, et al Cancer statistics for Hispanics, 2003. CA Cancer J Clin. 2003;53(4):208–26. Epub 2003/08/20. . 12924775 10.3322/canjclin.53.4.208

[3] Rosenblatt KA, Carter JJ, Iwasaki LM, Galloway DA, Stanford JL. Serologic evidence of human papillomavirus 16 and 18 infections and risk of prostate cancer. Cancer Epidemiol Biomarkers Prev. 2003;12(8):763–8. Epub 2003/08/15. . 12917208

[4] Somech R, Amariglio N, Spirer Z, Rechavi G. Genetic predisposition to infectious pathogens: a review of less familiar variants. Pediatr Infect Dis J. 2003;22(5):457–61. Epub 2003/06/07. 10.1097/01.inf.0000068205.82627.55 . 12792391

[5] Strathdee SA, Veugelers PJ, Moore PS. The epidemiology of HIV-associated Kaposi’s sarcoma: the unraveling mystery. AIDS. 1996;10 Suppl A:S51–7. Epub 1996/01/01. . 8883610

[6] Cesarman E, Knowles DM. Kaposi’s sarcoma-associated herpesvirus: a lymphotropic human herpesvirus associated with Kaposi’s sarcoma, primary effusion lymphoma, and multicentric Castleman’s disease. Semin Diagn Pathol. 1997;14(1):54–66. Epub 1997/02/01. . 9044510

[7] Toth Z, Brulois K, Jung JU. The chromatin landscape of Kaposi’s sarcoma-associated herpesvirus. Viruses. 2013;5(5):1346–73. Epub 2013/05/24. 10.3390/v5051346 . 23698402 PMC3712311

[8] Lukac DM, Kirshner JR, Ganem D. Transcriptional activation by the product of open reading frame 50 of Kaposi’s sarcoma-associated herpesvirus is required for lytic viral reactivation in B cells. J Virol. 1999;73(11):9348–61. Epub 1999/10/09. . 10516043 10.1128/jvi.73.11.9348-9361.1999 PMC112969

[9] Guito J, Lukac DM. KSHV Rta Promoter Specification and Viral Reactivation. Front Microbiol. 3:30 Epub 2012/02/22. 10.3389/fmicb.2012.00030 . 22347875 PMC3278982

[10] Lin SF, Robinson DR, Miller G, Kung HJ. Kaposi’s sarcoma-associated herpesvirus encodes a bZIP protein with homology to BZLF1 of Epstein-Barr virus. J Virol. 1999;73(3):1909–17. Epub 1999/02/11. . 9971770 10.1128/jvi.73.3.1909-1917.1999 PMC104432

[11] Izumiya Y, Lin SF, Ellison T, Chen LY, Izumiya C, Luciw P, et al Kaposi’s sarcoma-associated herpesvirus K-bZIP is a coregulator of K-Rta: physical association and promoter-dependent transcriptional repression. J Virol. 2003;77(2):1441–51. Epub 2002/12/28. 10.1128/JVI.77.2.1441-1451.2003 . 12502859 PMC140808

[12] Liao W, Tang Y, Lin SF, Kung HJ, Giam CZ. K-bZIP of Kaposi’s sarcoma-associated herpesvirus/human herpesvirus 8 (KSHV/HHV-8) binds KSHV/HHV-8 Rta and represses Rta-mediated transactivation. J Virol. 2003;77(6):3809–15. Epub 2003/03/01. 10.1128/JVI.77.6.3809-3815.2003 . 12610155 PMC149497

[13] Wang Y, Sathish N, Hollow C, Yuan Y. Functional characterization of Kaposi’s sarcoma-associated herpesvirus open reading frame K8 by bacterial artificial chromosome-based mutagenesis. J Virol. 85(5):1943–57. Epub 2010/12/17. 10.1128/JVI.02060-10 . 21159864 PMC3067771

[14] Kato-Noah T, Xu Y, Rossetto CC, Colletti K, Papouskova I, Pari GS. Overexpression of the kaposi’s sarcoma-associated herpesvirus transactivator K-Rta can complement a K-bZIP deletion BACmid and yields an enhanced growth phenotype. J Virol. 2007;81(24):13519–32. Epub 2007/10/05. 10.1128/JVI.00832-07 . 17913803 PMC2168825

[15] Ellison TJ, Izumiya Y, Izumiya C, Luciw PA, Kung HJ. A comprehensive analysis of recruitment and transactivation potential of K-Rta and K-bZIP during reactivation of kaposi’s sarcoma-associated herpesvirus. Virology. 2009;387(1):76–88. Epub 2009/03/10. 10.1016/j.virol.2009.02.016 . 19269659 PMC4327937

[16] Wu FY, Wang SE, Tang QQ, Fujimuro M, Chiou CJ, Zheng Q, et al Cell cycle arrest by kaposi’s sarcoma-associated herpesvirus replication-associated protein is mediated at both the transcriptional and posttranslational levels by binding to CCAAT/enhancer-binding protein alpha and p21(CIP-1). J Virol. 2003;77(16):8893–914. Epub 2003/07/30. 10.1128/JVI.77.16.8893-8914.2003 . 12885907 PMC167214

[17] Liu D, Wang Y, Yuan Y. kaposi’s Sarcoma-Associated Herpesvirus K8 Is an RNA Binding Protein That Regulates Viral DNA Replication in Coordination with a Noncoding RNA. J Virol. 2018 Epub 2018/01/13. 10.1128/JVI.02177-17 . 29321307 PMC5972905

[18] Guito J, Lukac DM. KSHV Rta Promoter Specification and Viral Reactivation. Front Microbiol. 2012;3:30 Epub 2012/02/22. 10.3389/fmicb.2012.00030 . 22347875 PMC3278982

[19] Kaul R, Verma SC, Murakami M, Lan K, Choudhuri T, Robertson ES. Epstein-Barr virus protein can upregulate cyclo-oxygenase-2 expression through association with the suppressor of metastasis Nm23-H1. J Virol. 2006;80(3):1321–31. Epub 2006/01/18. 10.1128/JVI.80.3.1321-1331.2006 . 16415009 PMC1346972

[20] Machanick P, Bailey TL. MEME-ChIP: motif analysis of large DNA datasets. Bioinformatics. 27(12):1696–7. Epub 2011/04/14. 10.1093/bioinformatics/btr189 . 21486936 PMC3106185

[21] Gupta S, Stamatoyannopoulos JA, Bailey TL, Noble WS. Quantifying similarity between motifs. Genome Biol. 2007;8(2):R24 Epub 2007/02/28. 10.1186/gb-2007-8-2-r24 . 17324271 PMC1852410

[22] Lai E, Clark KL, Burley SK, Darnell JE Jr. Hepatocyte nuclear factor 3/fork head or "winged helix" proteins: a family of transcription factors of diverse biologic function. Proc Natl Acad Sci U S A. 1993;90(22):10421–3. Epub 1993/11/15. . 8248124 10.1073/pnas.90.22.10421 PMC47788

[23] Liao W, Tang Y, Kuo YL, Liu BY, Xu CJ, Giam CZ. kaposi’s sarcoma-associated herpesvirus/human herpesvirus 8 transcriptional activator Rta is an oligomeric DNA-binding protein that interacts with tandem arrays of phased A/T-trinucleotide motifs. J Virol. 2003;77(17):9399–411. Epub 2003/08/14. 10.1128/JVI.77.17.9399-9411.2003 . 12915555 PMC187432

[24] Palmeri D, Carroll KD, Gonzalez-Lopez O, Lukac DM. kaposi’s sarcoma-associated herpesvirus Rta tetramers make high-affinity interactions with repetitive DNA elements in the Mta promoter to stimulate DNA binding of RBP-Jk/CSL. J Virol. 2011;85(22):11901–15. Epub 2011/09/02. 10.1128/JVI.05479-11 . 21880753 PMC3209305

[25] Chen J, Ye F, Xie J, Kuhne K, Gao SJ. Genome-wide identification of binding sites for kaposi’s sarcoma-associated herpesvirus lytic switch protein, RTA. Virology. 2009;386(2):290–302. Epub 2009/02/24. 10.1016/j.virol.2009.01.031 . 19233445 PMC2663009

[26] Ziegelbauer J, Grundhoff A, Ganem D. Exploring the DNA binding interactions of the kaposi’s sarcoma-associated herpesvirus lytic switch protein by selective amplification of bound sequences in vitro. J Virol. 2006;80(6):2958–67. Epub 2006/02/28. 10.1128/JVI.80.6.2958-2967.2006 . 16501105 PMC1395432

